# Urinary NMR Profiling in Pediatric Acute Kidney Injury—A Pilot Study

**DOI:** 10.3390/ijms21041187

**Published:** 2020-02-11

**Authors:** Claudia Muhle-Goll, Philipp Eisenmann, Burkhard Luy, Stefan Kölker, Burkhard Tönshoff, Alexander Fichtner, Jens H. Westhoff

**Affiliations:** 1 Karlsruhe Institute of Technology, Institute for Biological Interfaces 4, P.O. Box 3640, 76021 Karlsruhe, Germany; burkhard.luy@kit.edu; 2 Karlsruhe Institute of Technology, Institute of Organic Chemistry, Fritz-Haber-Weg 6, 76131 Karlsruhe, Germany; philipp-michael.eisenmann@kit.edu; 3 Division of Pediatric Neurology and Metabolic Medicine, University Children’s Hospital Heidelberg, Im Neuenheimer Feld 430, 69120 Heidelberg, Germany; stefan.koelker@med.uni-heidelberg.de; 4 Department of Pediatrics I, University Children’s Hospital Heidelberg, Im Neuenheimer Feld 430, 69120 Heidelberg, Germany; burkhard.toenshoff@med.uni-heidelberg.de (B.T.); alexander.fichtner@med.uni-heidelberg.de (A.F.)

**Keywords:** acute kidney injury, metabolomics, urine, NMR spectroscopy, multivariate analysis

## Abstract

Acute kidney injury (AKI) in critically ill children and adults is associated with significant short- and long-term morbidity and mortality. As serum creatinine- and urine output-based definitions of AKI have relevant limitations, there is a persistent need for better diagnostics of AKI. Nuclear magnetic resonance (NMR) spectroscopy allows for analysis of metabolic profiles without extensive sample manipulations. In the study reported here, we examined the diagnostic accuracy of NMR urine metabolite patterns for the diagnosis of neonatal and pediatric AKI according to the Kidney Disease: Improving Global Outcomes (KDIGO) definition. A cohort of 65 neonatal and pediatric patients (0–18 years) with established AKI of heterogeneous etiology was compared to both a group of apparently healthy children (*n* = 53) and a group of critically ill children without AKI (*n* = 31). Multivariate analysis identified a panel of four metabolites that allowed diagnosis of AKI with an area under the receiver operating characteristics curve (AUC-ROC) of 0.95 (95% confidence interval 0.86–1.00). Especially urinary citrate levels were significantly reduced whereas leucine and valine levels were elevated. Metabolomic differentiation of AKI causes appeared promising but these results need to be validated in larger studies. In conclusion, this study shows that NMR spectroscopy yields high diagnostic accuracy for AKI in pediatric patients.

## 1. Introduction

Acute kidney injury (AKI) is associated with poor outcomes, including increased morbidity (e.g., days on ventilator, length of hospital and intensive care unit stay) and mortality, and an increased risk for the development of chronic kidney disease [1,2,3]. The prevalence of neonatal and pediatric AKI among critically ill and high-risk cohorts is high, and AKI incidence is still increasing. Based on recent consensus definitions, AKI occurs in 27% and 29.9% of patients of the pediatric (PICU) and neonatal intensive care unit (NICU), respectively [4,5]. Remarkably, in non-critically ill hospitalized children and adolescents, AKI incidence is still as high as 5% [6]. The current gold standard for the diagnosis of AKI relies upon serum creatinine and urine output measurements, both of which, however, reveal relevant drawbacks. As such, serum creatinine is a late and indirect marker of reduced glomerular filtration rate that does not allow for differentiation of the specific cause of renal impairment. Urine output measurements, on the other hand, demand longer-term evaluations and are influenced, e.g., by diuretic medication. Of note, an earlier and more specific identification of patients with AKI and especially of those who are at highest risk for adverse outcome can influence physicians’ decision-making and medical treatment and may ultimately improve patient outcome.

In recent years, several urinary proteins including neutrophil gelatinase-associated lipocalin (NGAL), kidney injury molecule-1 (KIM-1), tissue inhibitor of metalloprotease-2 (TIMP-2), insulin-like growth factor-binding protein 7 (IGFBP7), and others have been proposed as useful biomarkers for early diagnosis, differentiation, and/or prediction of patient outcome in adult and pediatric AKI [7,8,9,10]. Unfortunately, the specificity of the above protein biomarkers for kidney injury and their clinical performance are insufficient for clinical implementation [11,12]. Hence, there is an unmet need for novel markers of AKI alone or in combination that i) improve patient risk stratification, ii) optimize early and precise detection of renal damage, iii) enable an early etiological classification of AKI, iv) monitor and target clinical management, and v) predict clinical outcome following AKI.

Untargeted metabolomics provides a functional fingerprint of the physiological and pathophysiological state of an organism and can be used both for pattern recognition and metabolite identification [13]. Nuclear magnetic resonance (NMR) and gas chromatography or liquid chromatography, together with mass spectrometry, are generally used to separate and identify metabolites. Metabolic approaches, i.e., by mass spectrometry, have been adopted to uncover new small molecule biomarkers or biochemical mechanisms as well as signaling pathways in chronic kidney disease, diabetic nephropathy, AKI, renal cancer, kidney transplantation, and polycystic kidney diseases [14,15]. These studies were performed in either rodents or humans and investigated blood, urine, or kidney tissue samples. In an experimental pig model of sepsis-induced AKI, NMR-based metabolomics was a potentially useful tool for biomarker identification [16]. Characteristic urine NMR spectra were also demonstrated in murine renal ischemia-reperfusion injury models [17,18]. Archdekin et al. using mass spectrometry of urine samples demonstrated the potential of a urine metabolite classifier to detect non-rejection kidney injury in pediatric kidney transplant patients and non-invasively discriminated non-rejection kidney injury from rejection [19]. While metabolomic profiles have been investigated in prematurity, low birth weight neonates, perinatal asphyxia, pediatric respiratory and neurological diseases, gastrointestinal diseases and inborn errors of metabolism, only very few clinical studies have been published that investigated the application of a metabolomic approach in pediatric AKI [20,21].

This prompted us to investigate the accuracy of NMR-based urine metabolomics for the diagnosis of AKI in a pilot cohort study of neonates and children with established Kidney Disease: Improving Global Outcomes (KDIGO) AKI of heterogeneous etiology. We further aimed to investigate if metabolomic fingerprints and biomarkers allow for a differentiation of specific AKI subtypes.

## 2. Results

### 2.1. Characteristics of the Study Population

Subject characteristics are shown in Table 1. As the urine heavily reflects environmental influences, two different control groups were included into the study in order to reduce the risk of a selection bias. While the first control group consisted of apparently healthy children (“healthy controls”), the second control group comprised neonatal and pediatric ICU patients without AKI (“non-AKI patients”). In brief, there was no significant difference regarding age, gender, proportion of neonates, and body mass index (BMI) standard deviation score (SDS) between AKI patients, non-AKI patients, and healthy controls. The etiology of AKI was heterogeneous including both prerenal and intrinsic causes. The more frequent AKI etiologies included dehydration (*n* = 15), hemolytic uremic syndrome (*n* = 13), septic shock (*n* = 12), perinatal asphyxia (*n* = 7), hemodynamic instability (*n* = 4), and interstitial nephritis (*n* = 4). Serum creatinine on study enrollment was significantly (*p* < 0.001) higher in AKI patients compared to non-AKI patients and, accordingly, estimated creatinine clearance (eCCl) was significantly reduced (*p* < 0.001).

### 2.2. Nuclear Magnetic Resonance (NMR) Spectroscopy Analysis

Ten typical spectra of the urines of AKI patients, non-AKI patients, and healthy controls, respectively, are shown in Supplemental Figure S1. Whereas spectra of healthy children had a uniform appearance, corresponding to the typical urine spectra of healthy people [22], AKI spectra revealed a different picture. Not only did the spectra show increased intensities due to the reduced urine volume, but metabolite composition varied between the different individuals. In addition, some AKI spectra revealed a background of broad unresolved resonances most likely originating from large protein or lipid/steroid signals. Non-AKI patient spectra were in-between the two other groups, showing variation in their metabolite composition, but not to the same extent as the AKI spectra. Because of the observed spectral diversity, we performed variable bucketing and selected preferably those peaks that were common among all three groups.

### 2.3. Multivariate Analysis

**Figure 1:**
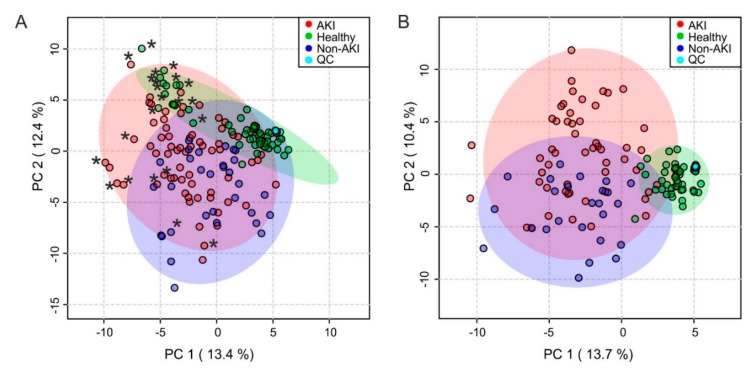
Principle component analysis of the urine spectral bucket table. Variable bucketing was employed leading to 129 buckets. Metabolites were assigned, whenever possible, based on comparison with database entries or literature and were confirmed by spiking experiments. (**A**) All spectra (AKI patients, *n* = 65; healthy controls, *n* = 53; non-AKI patients, *n* = 31; QC, *n* = 15), * denotes neonatal spectra, (**B**) neonatal spectra excluded (AKI patients, *n* = 53; healthy controls, *n* = 42; non-AKI patients, *n* = 25; QC, *n* = 15). Outliers were not detectable. AKI, acute kidney injury; QC, quality control.

When performing a principle component analysis (PCA), the AKI group could be clearly separated from the healthy control group (Figure 1A). However, the separation between the AKI and the non-AKI group was less apparent. Principal components 1 (PC1) and 2 (PC2) explained 13.4% and 12.4% of the variation. Quality control (QC) samples clustered tightly within the healthy control group and showed that measurements were highly reproducible. Neonatal spectra appeared to have more negative PC1 values. Especially among the healthy control group, neonatal spectra clustered in the upper left corner. Thus, the subsequent analyses were performed twice, with neonates either included or excluded. When neonates were excluded from the analysis (12 AKI patients, 11 healthy control subjects, six non-AKI control patients), separation was more pronounced (Figure 1B).

**Figure 2:**
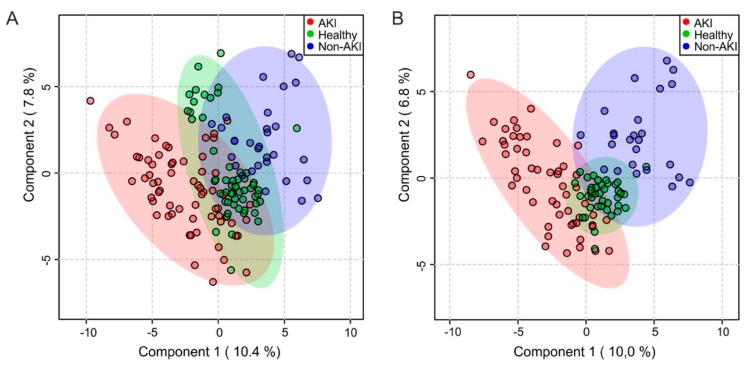
Partial least squares discriminant analysis of the urine spectral bucket table. Neonatal spectra (**A**) included (AKI patients, *n* = 65; healthy controls, *n* = 53; non-AKI patients, *n* = 31), (**B**) excluded (AKI patients, *n* = 53; healthy controls, *n* = 42; non-AKI patients, *n* = 25). AKI, acute kidney injury.

Partial least squares discriminant analysis (PLS-DA) was performed to separate within group variation from class-related differentiation (Figure 2A). Quality control parameters showed that AKI patients could be reliably separated from the control group (quality of prediction Q^2^: 0.55, explained variance R^2^: 0.76). Omitting neonatal samples (Figure 2B) only slightly improved the separation and predictive power (Q^2^: 0.64, R^2^: 0.82). A 1000-fold permutation analysis confirmed that this result was not fortuitous in both cases (*p* < 0.001). Separation into prerenal and intrinsic causes of AKI based on previously published criteria [23] was explored, but did not lead to improved clustering in the PCA or PLS-DA (Supplemental Figure S2) and was consequently abandoned.

As mentioned before, non-AKI patient spectra separated less well from AKI spectra than healthy control spectra. We questioned whether the analysis preferentially selected general traits of morbidity like markers of increased catabolic pathways or drug metabolism. To rule out this argument, we performed both PCA and PLS-DA in healthy controls and non-AKI controls (Supplemental Figure S3). Metabolites that separated the two groups were identified in a PLS-DA (Supplemental Table S1) and those with variable importance in projection (VIP) values higher than 1.5 were excluded in the following analyses. For further analyses, the healthy control group and the non-AKI patient group were combined. This led to a visibly better separation already in a PCA (Supplemental Figure S4A). A PLS-DA model could be obtained with three components and quality parameters Q^2^ of 0.66 and R^2^ of 0.79 (Supplemental Figure S4B, Table 2). Omitting neonates further improved the separation between the groups to a Q^2^ value of 0.73 and a R^2^ value of 0.89 (Supplemental Figure S5A and S5B, Table 2). The VIP plot (Supplemental Figures S4C and S5C) revealed that citrate, bile acid signals, leucine, valine, general lipid signals, cis-aconitic acid, formate, as well as non-assigned aliphatic (unk_al/x) compounds were mainly responsible for the separation.

### 2.4. Biomarker Analysis

To analyze whether the spectral information or a combination of several metabolites could be used as biomarker, 20% of randomly selected spectra (11 AKI, six non-AKI, and eight healthy control spectra) were not used for initial analysis and served as validation cohort. Neonates were excluded as the previous analysis had shown that their spectral characteristics differ to a certain degree. We developed a diagnostic model based on the metabolites with the highest VIP values in the PLS-DA analysis (Supplemental Figure S5C, Table 3). They all demonstrated AUC-ROC values > 0.70 and confidence intervals that did not cross 0.5. Yet a logistic regression model with these 15 markers did not converge. Thus, we successively reduced the number of potential markers based on the calculated z values and Pr(>|z|) to a set of four markers: Citrate with its resonance at 2.69 ppm, leucine (0.96 ppm), valine with its resonance at 1.00 ppm, and bile acid (0.69–0.81 ppm). 

We obtained a regression equation with a best threshold (or cut-off) for the predicted *p* (= Pr(y=1|x)) of 0.34 (Table 4A).

**Figure 3:**
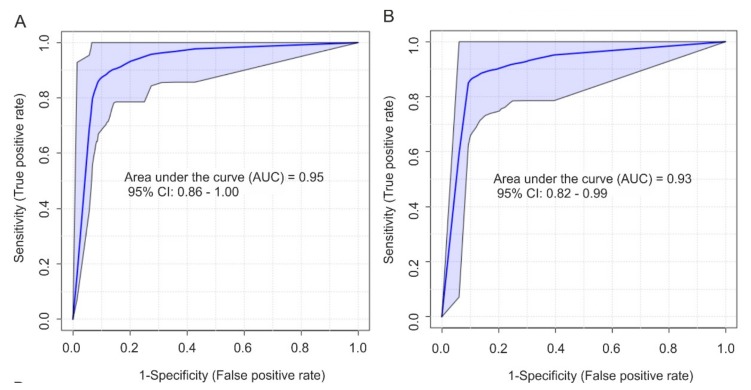
ROC curve for the diagnostic accuracy of selected metabolites from NMR spectroscopy for the diagnosis of KDIGO AKI obtained from a linear regression analysis using (**A**) four selected features (citrate (2.69 ppm), leucine (0.96 ppm), valine (1.00 ppm), and bile acid (0.69–0.81 ppm)) and (**B**) six selected features (citrate (2.69 ppm), leucine (0.96 ppm), valine (1.00 ppm), bile acid (0.69–0.81 ppm), unk_al1 (3.16 ppm), and unk_al28 (2.12–2.17 ppm)). AKI, acute kidney injury; KDIGO, Kidney Disease: Improving Global Outcomes; NMR, nuclear magnetic resonance; ROC, receiver operating characteristic.

*p*-values above this threshold classified samples as AKI. The area under the receiver operating characteristics curve (AUC-ROC) was 0.95 with a 95% confidence interval of 0.86–1.00 (Figure 3A). When the classification performance was tested on the validation cohort, one AKI test sample (out of 11) was wrongly classified, but no control spectra. A modified marker set including two additional unidentified aliphatic compounds, one with a resonance at 3.16 ppm (unk_al1) and one overlapping with glutamine (2.12–2.17 ppm, unk_al28), classified all spectra of the validation set correctly, but had a slightly lower AUC-ROC value of 0.93 (95% CI: 0.82–0.99) (Table 4B and Figure 3B). Details about each feature are listed in Table 4A,B.

### 2.5. Differentiation of Acute Kidney Injury (AKI) Etiologies

**Figure 4:**
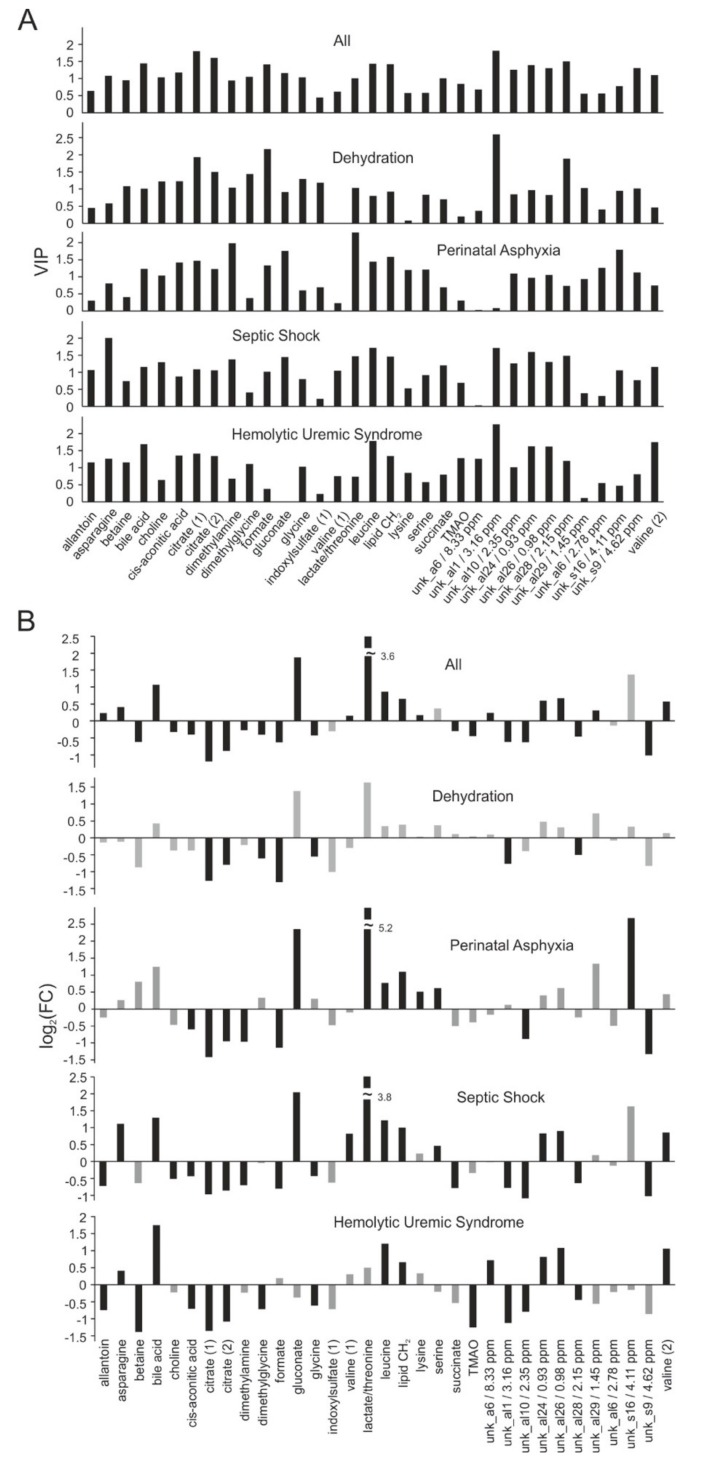
(**A**) Overview of discriminant identified metabolites according to variable importance in projection (VIP) values analyzed separately for each etiology. VIP values were obtained from a PLS-DA analysis. Only metabolite buckets are shown that had VIP values > 1.1 for at least one of the different groups. (**B**) The respective fold changes (log_2_(FC)) for the same metabolites are given for comparison. Black bars denote fold changes with an associated *p*-value < 0.01, grey bars those with a *p*-value > 0.01. PLS-DA, partial least squares discriminant analysis.

We next analyzed whether NMR spectroscopy allows for etiologic differentiation of underlying AKI causes (Figure 4). Four AKI subgroups had sufficient patient numbers for statistical analysis (*n* > 5). Here we used a reduced set of buckets and excluded all buckets of the original set that had variable importance in projection (VIP) values < 1.1 when the AKI group was compared against the combined control group. This led to a reduced set of 48 buckets. The logic behind this choice was to remove potential noise as the number of variables by far exceeded the number of spectra.

We performed PLS-DA with each of the subgroups (i.e., dehydration, hemolytic uremic syndrome, septic shock, perinatal asphyxia) against the combined control group to search for distinct group specific patterns. Quality control values were good for two subgroups (septic shock and hemolytic uremic syndrome) and acceptable for the dehydration subgroup (Table 2). A moderate Q^2^-value and a high empirical *p*-value for a 1000-fold permutation test showed that the subgroup perinatal asphyxia was less well identifiable with the employed metabolite set.

Figure 4 displays the VIP values of the most important metabolites (VIP > 1.1 in at least one group) together with their fold change calculated for each subgroup. The VIP patterns (Figure 4A) showed that in addition to metabolite buckets that were important for general AKI identification in all AKI subjects, AKI subtype-specific metabolite buckets appeared to be identifiable. Fold changes analysis displayed an even more distinct picture (Figure 4B). A fold change in citrate was common in all groups. Others seemed to be specific for the underlying AKI etiology. For example, gluconate and lactate-to-threonine ratio had large fold changes in perinatal asphyxia and septic shock, but were less informative for dehydration and hemolytic uremic syndrome.

## 3. Discussion

Several metabolomic studies investigating various renal diseases have been published in the past decade, however, metabolomics data on pediatric AKI is scarce (for an overview see [14,15,24]). In the present study, ^1^H-NMR profiling was applied to urinary samples of neonatal and pediatric patients with established AKI and compared to healthy children and to hospitalized children without AKI. By multivariate analysis, we identified a panel of metabolites that enabled AKI diagnosis by yielding an AUC-ROC of 0.95. The identified metabolites that enabled the diagnosis of AKI (KDIGO) included, among several unknown compounds, increases in leucine, valine, bile acid and decreases in citrate (Figure 4B). In addition, in our rather small pilot study population NMR-fingerprints seemed to allow for differentiation between different AKI etiologies.

A crucial point in our analysis was the selection of the control group. The AKI cohort was very heterogeneous with respect to the underlying cause of AKI, and patients were treated with a variety of medications. As urine reflects all these impacts as well as nutritional influences, comparing the AKI children only to a healthy control cohort would enhance the risk for the selection of markers not related to AKI but rather to general morbidity. Creatinine may be such an example, as it is not only used to determine renal clearance but is also a marker of muscle mass. For that reason, we decided to further include a control group of critically ill ICU patients without AKI that were also exposed to diverse medical treatments. PCA and PLS-DA analysis proved that this grouping was possible and that AKI spectra showed their own distinct pattern. Moreover, in this way we identified metabolites like mannitol, lactose, and creatinine as crucial for the general distinction between healthy children and hospitalized patients without AKI. Mannitol and lactose are applied as additive drug components, whereas creatinine may be a common sign of catabolic metabolism or a sign of a minor reduction in renal clearance (Supplemental Table S1). By omitting these metabolites, we are confident that the distinction of AKI from non-AKI patients was not based on common medication or general signs of morbidity. Strikingly, some markers that were excluded from analysis after comparison of healthy control and non-AKI patient control spectra (hippurate, indoxylsulfate, creatinine) were among those that were previously classified as markers for AKI in animal models [25,26,27]. However, in animal models, only healthy untreated animals served as control. This does not allow a distinction between general signs of morbidity and specific ones for AKI.

Previous human AKI studies resulted in the identification of a variety of potential metabolite biomarkers [14,24]. Interestingly, almost no overlap exists between the respective metabolites [28,29,30,31,32,33,34] and the metabolites identified in our study. For example, Beger et al. identified homovanillinic acid sulfate, a dopamine metabolite as the most important marker in the urine of children who developed AKI after cardiac surgery [21]. This finding was confirmed by Mercier et al. investigating the urine of neonates with AKI [29]. By contrast, aromatic compounds other than 3-indoxylsulfate and hippurate are not among the important metabolites in our study.

Animal studies present a somewhat more homogeneous picture of putative biomarkers. In the majority, AKI was induced in healthy animals through application of drugs or surgery [25,26,27,34,35,36,37,38]. Although the identified biomarkers rarely fully matched between the different studies, metabolites of the citric acid cycle (TCA) cycle, branched-chain amino acids, creatinine, hippurate, and 3-indoxylsulfate emerged as common markers of drug-induced AKI [25,26,27,34,35,36,37,38]. Some prospective markers in rodent models of drug-induced AKI (e.g., by cisplatin or gentamicin) were already altered in early phases of drug-induced nephrotoxicity [25]. Strikingly though, the metabolite selection mentioned above overlaps with the markers identified in our study. Especially citrate, leucine, and valine were among the most relevant metabolites responsible for AKI diagnosis and were main components of our developed logistic regression model.

Why do our results match better to studies on animals than on humans? Previous human studies generally examined very homogeneous groups of patients with respect to AKI etiology. In our study, AKI patients comprised a broad etiologic spectrum of AKI which is reflected by the diverse peak patterns in the aromatic area (Figure 1). This diversity may have helped in identifying a robust common denominator. Enhanced branched-chain amino acids and a reduction in citrate excretion has been reported previously, but for other nephropathies including chronic kidney disease, [39,40], diabetic nephropathy [41] and polycystic kidney disease [42]. They may be signs of early kidney damage, as a reduction of citrate can be a sign for mitochondrial dysfunction of the proximal tubular cell [24].

In fact, a reduction of TCA cycle metabolites as reflected in our study presumably mirrors tubular dysfunction due to inadequate energy supply. This dysfunction might originate from missing driving forces of the tubular cell membrane due to a lack of adenosine triphosphate (ATP) that ultimately impairs transcellular transport of dicarbonic acids by organic anion transporters (OAT), i.e., OAT4 [43]. The reason for the reduction of urinary formic acid in pediatric AKI patients can only be speculated on. Energy deficiency might cause a reduced outwardly directed electrochemical gradient for formate, a metabolite that was also significantly reduced in AKI patients. This, in consequence, is a driving force for tertiary active Cl- absorption via the Cl-/formate anion exchange transporter [44].

In our study, the percentage of spectra originating from neonates was 18.5% in the AKI group, 19.4% in the non-AKI patient group, and 20.8% in the healthy control group. Omitting these from the statistical analyses further improved the separation between the AKI group and the combined control group comprising both healthy children and critically ill ICU patients without AKI. Similar observations were made previously when investigating novel protein biomarkers of renal damage in mixed neonatal and pediatric cohorts [23]. This observation might be attributed, at least in part, to the relatively high percentage of low KDIGO AKI stages in neonates. As such, four neonates (33.3%) were classified as Stage 1 KDIGO AKI, three neonates (25%) were classified as Stage 2 and only five patients (41.7%) were classified as Stage 3 AKI. By contrast, 46 (86.8%) pediatric AKI patients fulfilled Stage 3 KDIGO AKI criteria, six (11.3%) fulfilled Stage 2 criteria and only one pediatric AKI patient (1.9%) fulfilled Stage 1 criteria. In addition, several metabolomics studies demonstrated that the urine of neonates shows characteristic differences compared to older children. Scalabre et al. stressed the differential impact of age, height, and weight in metabolomic studies on urinary metabolic profiles in children aged < 1 year [45]. Among the metabolites identified as markers of age, they also identified citrate as an important marker. In that study, it was correlated with an increase in weight and height, and showed a negative correlation with age like succinate, which was confirmed in two other studies [20,46,47]. In comparison, in our study AKI patients of all ages showed a decrease of citrate levels compared to non-AKI subjects. Other age-related metabolites identified in these studies showed no significant effect in our analysis. Thus, we are confident that in our study age-related effects were marginal compared to the metabolic changes caused by AKI.

There are several limitations to our study. First, the number of patients participating in our single-center pilot study was low, hence requiring larger population studies. Nevertheless, robust identification of AKI patients by ^1^H-NMR spectroscopy was feasible despite the wide variety of AKI etiologies. Second, due to the low number of study participants investigated, this study was not appropriate for analyzing the impact of AKI severity as reflected by KDIGO stage on NMR spectra. Third, the presented study primarily focuses on the diagnosis of established AKI using the serum creatinine- and urine output-based KDIGO AKI criteria as gold standard. However, due to a variety of reasons both parameters can be problematic for AKI diagnosis, especially in children. Future studies will have to deal with the implementation of ^1^H-NMR spectroscopy for early detection of imminent AKI, for differentiation of varying AKI subtypes and for therapy monitoring and prognosis of AKI outcome. In fact, early identification of patients at highest risk for AKI and for adverse outcome can help physicians in early decision-making, e.g., with respect to the timely insertion of dialysis catheters and initiation of renal replacement therapy.

## 4. Materials and Methods

### 4.1. Ethics Statement

The study was conducted in accordance with the Declaration of Helsinki and approved by the ethics committee of the Heidelberg Medical Faculty (Protocol S-133/2011, permission: 15 July 2011, amendment: 13 June 2016). Legal guardian of each patient gave written informed consent and, when appropriate, assent from the patient was obtained as well.

### 4.2. Study Design and Participants

A prospective pilot cohort study was conducted at the University Children’s Hospital Heidelberg. Patients aged 0 to 18 years who developed AKI during their hospital stay and patients who were referred to our Children’s Hospital with established AKI were enrolled in the study from October 2011 to March 2019. Of note, the study population has partly been published before [23,48,49]. Criteria for study exclusion were (i) prematurity, (ii) postrenal AKI as examined by initial renal ultrasound, and (iii) children undergoing cardiac surgery. AKI was classified either according to the Kidney Disease: Improving Global Outcomes (KDIGO) AKI definition [50] for pediatric patients or according to the modified KDIGO definition for neonatal patients aged ≤ 28 days of life [51]. When serum creatinine and urine output criteria resulted in different KDIGO stages, the higher stage was chosen. The revised Schwartz formula (k = 0.413 × height / serum creatinine) was used for calculation of eCCl [52]. Baseline serum creatinine was defined as last value within the previous three months before study enrollment. In the case of missing baseline data, the eCCl was assumed to be 120 mL per minute per 1.73 m^2^ in pediatric patients and 40 mL per minute per 1.73 m^2^ in neonates [4,48]. Control subjects without AKI were taken from two different cohorts [23]. While the “non-AKI patients” group (*n* = 31) consisted of neonatal and pediatric ICU patients without AKI, the “healthy controls” group (*n* = 53) comprised apparently healthy neonates, children, and adolescents aged 0–18 years. Diagnoses of inpatients without AKI were postoperative care (*n* = 25 including neurosurgery, *n* = 11; pediatric surgery, *n* = 6; maxillofacial surgery, *n* = 6; orthopedics, *n* = 1; otorhinolaryngology, *n* = 1), seizures (*n* = 1), respiratory diseases (*n* = 1), perinatal asphyxia (*n* = 1), infectious diseases (*n* = 2), and cardiovascular diseases (*n* = 1). Exclusion criteria for the healthy control group have been previously published [48]. Patients’ characteristics are given in Table 1.

### 4.3. Sample and Data Collection

Urine samples were collected immediately following KDIGO AKI diagnosis or after admission to our hospital. In case of anuria, urine samples were obtained after restoration of diuresis. Following centrifugation, the supernatants of the urine samples were frozen, stored at −80 °C and thawed prior to analysis. After thawing urine samples were centrifuged for 10 min at 15,871 g. A total of 500 µL of samples were mixed with 100 µL sodium phosphate buffer (150 mM final concentration in D_2_O, pH 7.2) containing 2 mM trimethylsilylpropanoic acid for spectral referencing. D_2_O was used as lock substance. Measurement of SCr was performed using an IDMS-traceable enzymatic method.

### 4.4. ^1^H-NMR Spectroscopy of Urine Samples

A 600 MHz Avance II spectrometer (Bruker Biospin, Rheinstetten, Germany) with a double resonance 5-mm BBI probe was used to acquire the ^1^H NMR spectra at 300 K. Temperature calibration was performed on a daily basis. Quality control (QC) samples consisting of urine aliquots of a healthy male volunteer were included every 10 h of measurement. One-dimensional spectra were acquired with the Bruker pulse sequence noesygppr1D using a relaxation delay of 10 s, 32 transients of 64 K data points, and a spectral width of 20 ppm. Water suppression was achieved through presaturation with a bandwidth of 25 Hz. Automatic phase- and baseline-correction was employed. Spectra were calibrated to the signal of TSP. Prior to Fourier transformation, spectra were multiplied with an exponential function with a 0.3 Hz line broadening factor. Topspin3.2 (Bruker Biospin, Rheinstetten, Germany) was used for data acquisition and processing.

Variable bucketing was employed leading to 129 buckets. Metabolites were assigned on the basis of comparison with data bases entries or literature whenever possible. Spiking experiments, where specific metabolites were added to a representative, already measured sample, were used to confirm the assignments in cases of doubt. Two windows were excluded: 4.74–4.84 ppm and 6.0–5.56 ppm, where water or urea resonances, respectively, appear. Missing values did not occur.

### 4.5. Statistical Analysis

As clinical data was non-normally distributed, median and interquartile range are presented. For statistical analysis of intergroup differences of numeric parameters, the Kruskal–Wallis test with post hoc Dunn’s test or the Mann–Whitney U-test were used. Categorical parameters were compared by Pearson’s Chi-squared test. For age-independent estimates, BMI was converted to SDS values, related to age- and gender-specific means and SD of European reference populations.

PCA and PLS-DA were performed with R (version 3.5.3) using the package MetaboanalystR and default parameters [53]. Probabilistic quotient normalization [54] was employed prior to statistical analysis using the healthy control samples as reference to take into account that the urine of AKI patients is as such more often concentrated leading to higher peak intensities. Data distribution showed that the data were skewed. To take this into account, data were log transformed and scaled to unit variance. To assess the quality of the resulting statistical models, 10-fold internal cross-validation as well as permutation tests were performed. To judge the predictive power of PLS-DA, quality parameters Q^2^ (quality of prediction) and R^2^ (explained variance) were calculated. In a second round, metabolites that separated healthy controls and non-AKI controls were identified in a PLS-DA (Supplemental Table S1) and those with VIP values higher than 1.5 (18 buckets) were excluded from the subsequent analyses.

Biomarker analysis was performed with the respective module of MetaboanalystR using PLS-DA for identification of a set of marker peaks, support vector machines, and logistic regression to assess the suitability of a reduced set for class prediction of unknown spectra and univariate ROC curve analysis for the calculation of individual metabolite AUC values.

For etiological analysis a reduced set of metabolites was used. All buckets were taken out that had variable importance in projection (VIP) values < 1.1 when the AKI group was compared against the combined control group. This led to a reduced set of 48 buckets. Metabolite fold changes between the groups were assessed with the non-parametric Mann-Whitney U-test using the Fold-Change Analysis Module of MetaboanalystR.

## 5. Conclusions

In conclusion, the present study underlines the value of 1H-NMR spectroscopy for the identification of novel biomarkers of renal damage in pediatric AKI. By use of this elegant technique, we were able to identify a panel of four metabolites that in linear regression analysis yielded an excellent accuracy for the diagnosis of AKI. In addition, detailed breakdown of signaling pathways being involved in the AKI cascade might further pave the way for the development of new therapeutic options for AKI.

## Figures and Tables

**Table 1:** Characteristics of the study population. Numeric data are presented as median with interquartile range (IQR) in parenthesis. Categorical data are presented as a number with the percentage in parenthesis. Statistical tests used for the individual parameters are described in the section Statistical Analysis. *p* < 0.05 was regarded as statistically significant. Abbreviations: AKI, acute kidney injury; BMI, body mass index; eCCl, estimated creatinine clearance; KDIGO, Kidney Disease: Improving Global Outcomes; SDS, standard deviation score.

Characteristic	AKI Patients (*n* = 65)	Non-AKI Patients (*n* = 31)	Healthy Controls (*n* = 53)	*p*-Value
Age (years)	3.0 (0.6 to 13.5)	1.0 (0.4 to 7.4)	6.0 (1.0 to 10.0)	0.21
Male Female	30 (46.2%) 35 (53.8%)	15 (48.4%) 16 (51.6%)	26 (49.1%) 27 (50.9%)	0.57
Neonates	12 (18.5%)	6 (19.4%)	11 (20.8%)	0.95
BMI SDS	−0.5 (−1.2 to 0.5)	−0.8 (−1.9 to 0.6)		0.25
AKI etiology				
Dehydration	15 (23.0%)			
Hemolytic uremic syndrome	13 (20.0%)			
Septic shock	12 (18.5%)			
Perinatal asphyxia	7 (10.8%)			
Hemodynamic	4 (6.2%)			
Interstitial nephritis	4 (6.2%)			
Other	10 (15.4%)			
Serum creatinine on study enrollment (mg/dL)	1.60 (0.9 to 3.5)	0.3 (0.2 to 0.5)		<0.001
eCCl on study enrollment (mL/min per 1.73 m^2^)	19.2 (11.0 to 42.0)	123.0 (80.9 to 170)		<0.001
KDIGO staging of AKI				
Stage 1	5 (7.7%)			
Stage 2	9 (13.8%)			
Stage 3	51 (78.5%)			
Proteinuria (g/L)	0.6 (0.2 to 1.5)	0.1 (0.0 to 0.1)		<0.001
Urinary protein-to-creatinine ratio (mg/g)	239 (48.6 to 955)	34.6 (24.6 to 49.3)		<0.001
Urinary leukocytes (per μL)	22.0 (4.5 to 93.0)	5.0 (0.0 to 26.5)		<0.01
Urinary erythrocytes (per μL)	15.0 (2.0 to 136.0)	5.0 (2.0 to 37.3)		<0.05
Squamous epithelium (per μL)	0.0 (0.0 to 1.5)	0.0 (0.0 to 0.0)		0.074
C-reactive protein (mg/L)	30.9 (7.5 to 98.8)	2.4 (0.0 to 19.0)		0.001
Renal replacement therapy	27 (41.5%)	0 (0%)	0 (0%)	<0.001
3-month mortality	11 (16.9%)	0 (0%)	0 (0%)	<0.05

Maximum KDIGO stage during the clinical course was Stage 3 in 51 patients (78,5%), Stage 2 in nine patients (13.8%), and Stage 1 in five patients (7.7%). Proteinuria (*p* < 0.001), urinary protein-to-creatinine-ratio (*p* < 0.001), urinary leukocytes (*p* = 0.002), urinary erythrocytes (*p* < 0.05), and C-reactive protein (*p* = 0.001) were significantly increased in AKI patients compared to non-AKI patients. Twenty-seven AKI patients (41.5%) required renal replacement therapy during the clinical course, 11 AKI patients (16.9%) deceased within 3 months following study enrollment.

**Table 2:** Explained variance and predictability for AKI and subgroups when compared to the combined control group (non-AKI patients plus healthy control subjects).

Group	PLS-DA Parameters			
	No. of Components	Q^2^	R^2^	Permutation *: Empirical *p*-Value
AKI (total)	3	0.63	0.78	< 0.001
AKI (neonates excluded in AKI and CTL)	4	0.73	0.89	< 0.001
Dehydration	3	0.44	0.80	< 0.001
Perinatal asphyxia	2	0.26	0.61	0.159
Septic shock	4	0.73	0.95	< 0.001
Hemolytic uremic syndrome	4	0.69	0.90	< 0.001

* resulting from 1000 times permutation analysis.

**Table 3:** Top 15 metabolites according to PLS-DA of AKI compared to the combined control group (non-AKI patients plus healthy control subjects), neonatal spectra excluded. AKI, acute kidney injury; PLS-DA, partial least squares discriminant analysis.

Metabolite Importance Number	Metabolite	VIP Score	AUC *	95% CI **
1	Citrate1 ***	2.73	0.94	0.88–0.98
2	Bile acid	2.52	0.91	0.84–0.97
3	Citrate2 ***	2.50	0.92	0.85–0.96
4	Unk_al1	2.49	0.90	0.83–0.96
5	unk_al28	2.19	0.84	0.75–0.91
6	Leucine	2.13	0.83	0.73–0.90
7	unk_al24	2.07	0.83	0.74–0.92
8	unk_al26	2.07	0.82	0.73–0.91
9	Valine	1.89	0.79	0.70–0.89
10	unk_al10	1.86	0.80	0.70–0.89
11	cis-Aconitic acid	1.86	0.79	0.69–0.88
12	Formate	1.80	0.84	0.75–0.91
13	LipidCH_2_	1.65	0.82	0.72–0.90
14	Glycine	1.50	0.71	0.61–0.81
15	Asparagine	1.48	0.76	0.65–0.85

* ROC AUC values from univariate testing. ** 95% confidence interval was calculated using 500 bootstrappings. *** Citrate is characterized by two separate buckets, one for each proton of the degenerate CH_2_ group.

**Table 4:** Logistic regression model—summary of each feature: (**A**) With four, (**B**) with six features.

(**A**) **logit(*p*) = log(*p*/(1 − P)) = −0.667 − 2.723 citrate + 2.538 leucine − 3.42 valine + 3.164 bile acid**					
	**Estimate**	**Std. Error**	**z Value**	**Pr(>|z|)**	**Odds**
(Intercept)	−0.667	0.571	−1.167	0.243	-
citrate (2.69 ppm)	−2.723	0.83	−3.281	0.001	0.07
leucine	2.538	1.089	2.33	0.02	12.65
valine	−3.42	1.374	−2.489	0.013	0.03
bile acid	3.164	1.096	2.887	0.004	23.67
(**B**) **logit(P) = log(P/(1 − P)) = 0.034 − 3.193 citrate + 4.859 leucine − 6.191 valine + 4.207 bile acid - 2.694 unk_al1 − 2.489 unk_al28**					
	**Estimate**	**Std. Error**	**z value**	**Pr(>|z|)**	**Odds**
(Intercept)	0.034	0.998	0.034	0.973	-
citrate (2.69 ppm)	−3.193	1.682	−1.898	0.058	0.04
leucine	4.859	2.182	2.227	0.026	128.88
valine	−6.191	2.617	−2.366	0.018	0
bile acid	4.207	1.774	2.372	0.018	67.18
unk_al1/3.16 ppm	2.694	1.414	−1.906	0.057	0.07
unk_al28/2.15 ppm	−2.489	1.093	−2.278	0.023	0.08

## Supplementary Materials

The following are available online at https://www.mdpi.com/1422-0067/21/4/1187/s1, Figure S1: Ten representative spectra of each group, Figure S2: PCA of AKI classified into prerenal (AKI-pre) and intrinsic (AKI-intrin), Figure S3: PCA and PLS-DA of healthy control group vs. hospitalized patients without AKI (Non-AKI), Figure S4: PCA and PLS-DA after taking out buckets separating healthy control group from hospitalized patients without AKI, Figure S5: Same as Figure S4, with neonatal spectra excluded. Table S1: Variable importance in projection (VIP) –Values from PLS-DA of healthy control group vs. hospitalized patients without AKI (Non-AKI).

## Abbreviations

AKI	acute kidney injury
AUC	area under the curve
BMI	body mass index
eCCl	estimated creatinine clearance
KDIGO	Kidney Disease: Improving Global Outcomes
NICU	neonatal intensive care unit
NMR	nuclear magnetic resonance
PCA	principle component analysis
PICU	pediatric intensive care unit
PLS-DA	partial least squares discriminant analysis
QC	quality control
ROC	receiver operating characteristic
SDS	standard deviation score
VIP	variable importance in projection
