# Supplementary material for: Urinary NMR Profiling in Pediatric Acute Kidney Injury—A Pilot Study
Source: Int J Mol Sci. 2020 Feb 11;21(4):1187. doi: 10.3390/ijms21041187 (PMC7072839; doi:10.3390/ijms21041187)
Supplement: Supplementary file 1 [file ijms-21-01187-s001.pdf]

Urinary NMR profiling in pediatric acute kidney injury - a pilot study

Claudia Muhle-Goll[a,b]*, Philipp Eisenmann[b], Burkhard Luy[a,b], Stefan Kölker[c], Burkhard Tönshoff[d], Alexander Fichtner[d], Jens H. Westhoff[d]*

[a] Karlsruhe Institute of Technology, Institute for Biological Interfaces 4, P.O. Box 3640, 76021 Karlsruhe, Germany

[b] Karlsruhe Institute of Technology, Institute of Organic Chemistry, Fritz-Haber-Weg 6, 76131 Karlsruhe, Germany

[c] Division of Pediatric Neurology and Metabolic Medicine, University Children's Hospital Heidelberg, Im Neuenheimer Feld 430, 69120 Heidelberg, Germany

[d] Department of Pediatrics I, University Children's Hospital Heidelberg, Im Neuenheimer Feld 430, 69120 Heidelberg, Germany

Supplemental Information

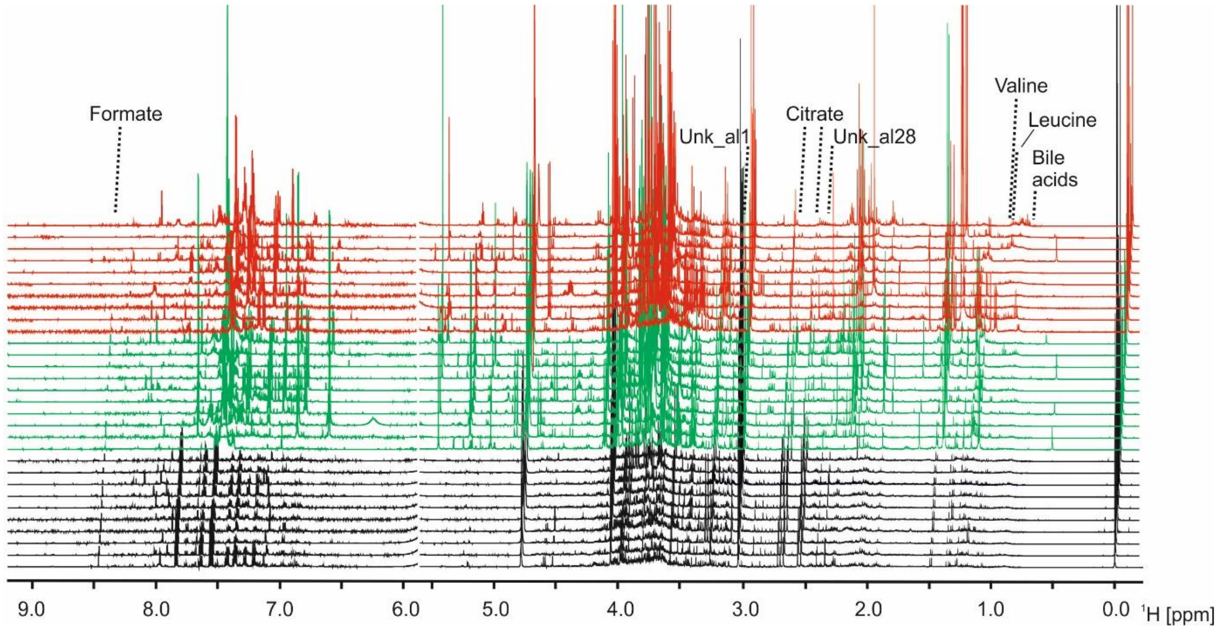

Figure S1. Ten representative spectra of each group: healthy control group – black, hospitalized patients without AKI – green, AKI – red.

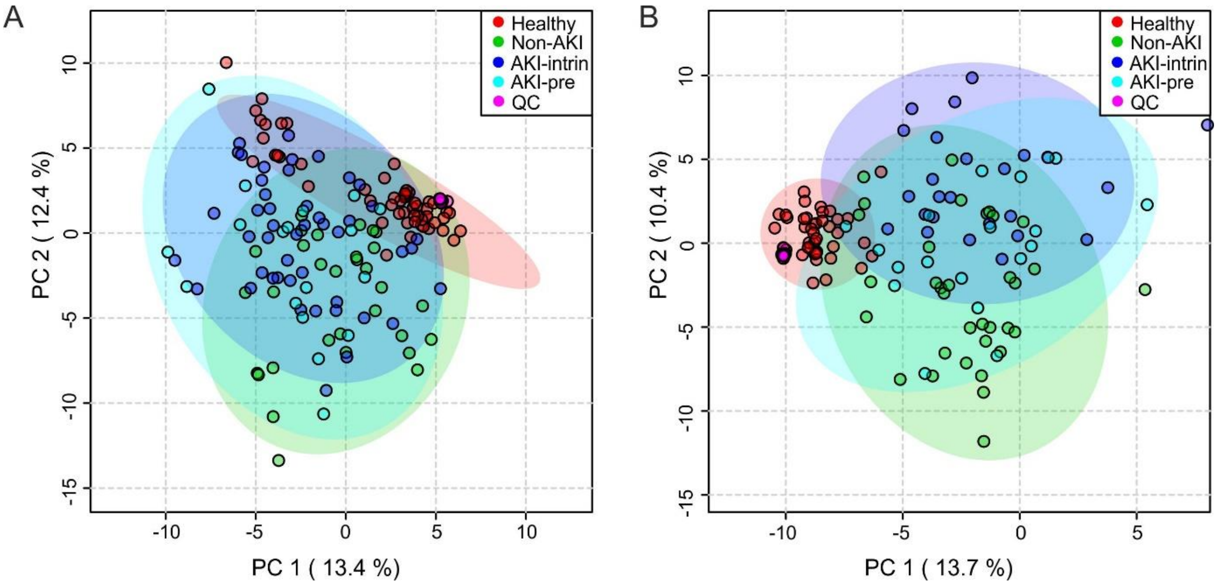

Figure S2: Principal component analysis. AKI classified into prerenal (AKI-pre) and intrinsic (AKI-intrin). A) Neonates included into study cohort, B) neonates excluded. Healthy – healthy control group. Non-AKI – hospitalized patients without AKI, QC – quality control.

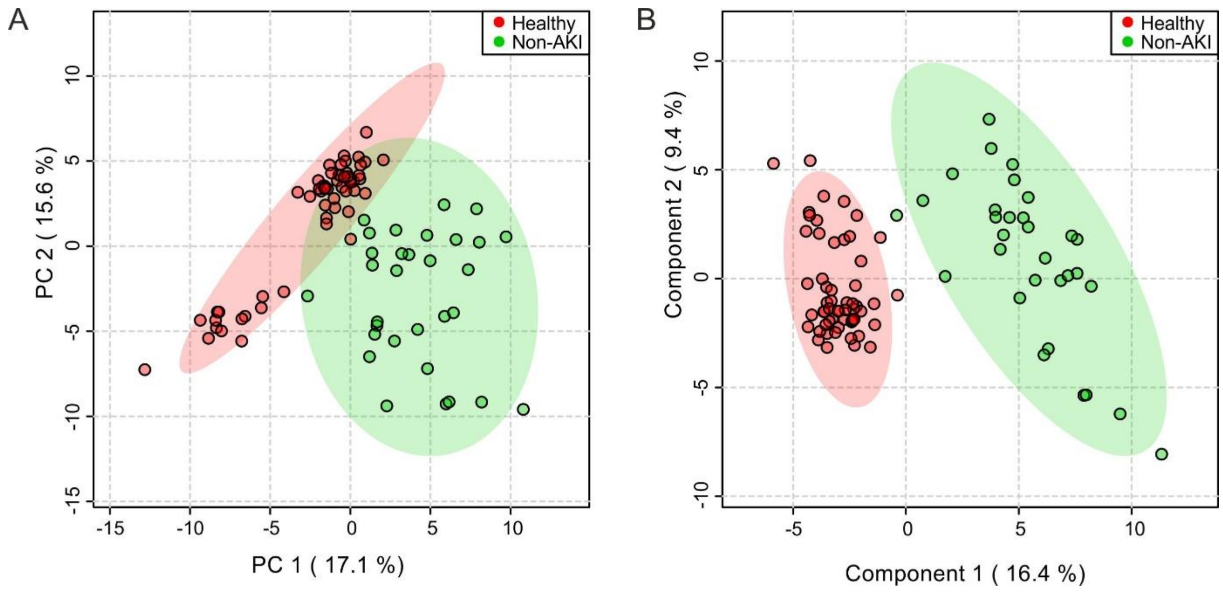

Figure S3: Statistical analysis of healthy control group vs. hospitalized patients without AKI (Non-AKI): A) Principal component analysis. B) Partial least squares discriminant analysis. R^2 0.97, Q^2 0.87, 5 components, p value in permutation < 0.001.

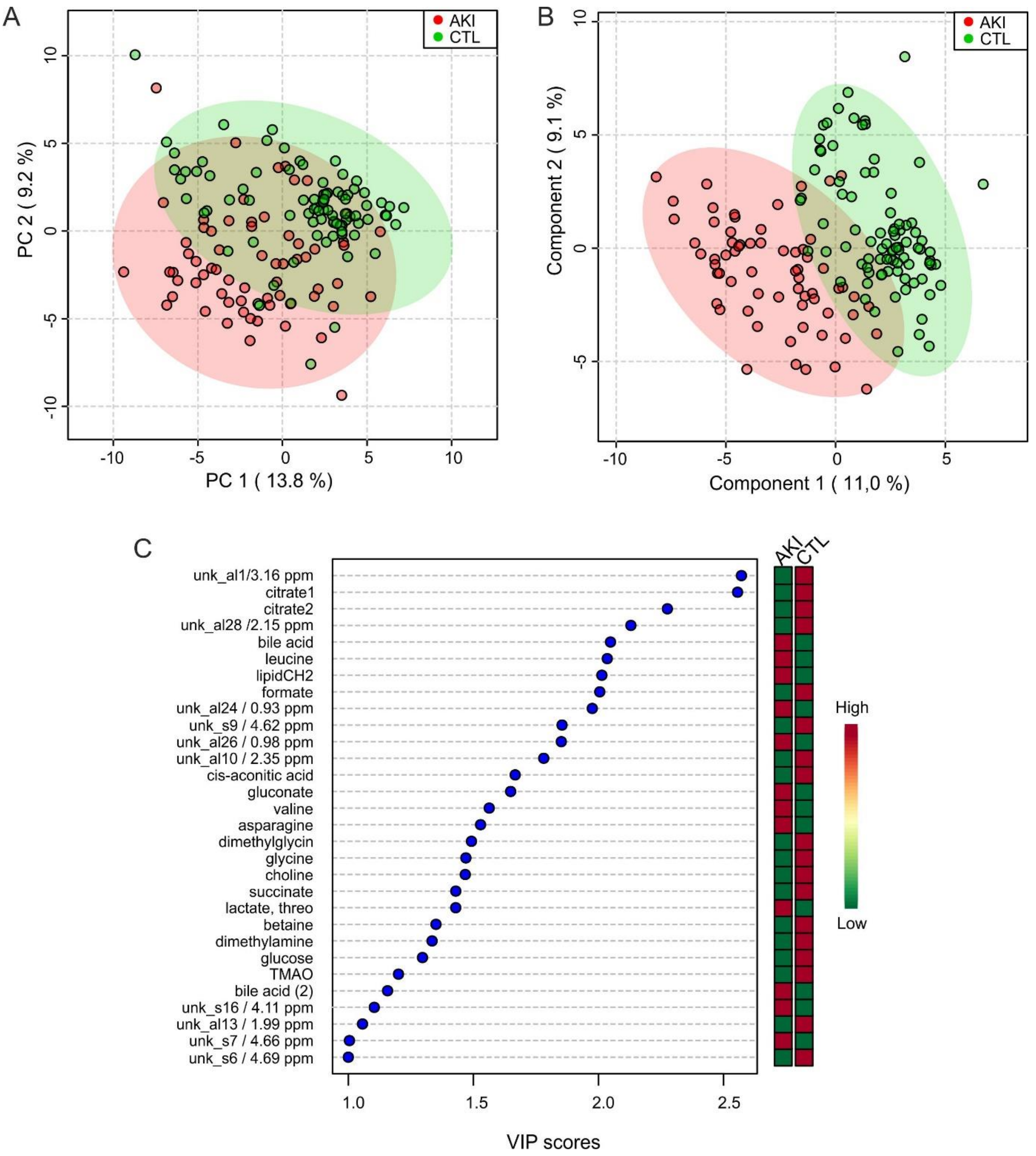

Figure S4. AKI vs CTL after taking out buckets separating healthy control group from hospitalized patients without AKI. Non-AKI and healthy control groups were joined in one group CTL. A) Principal component analysis. B) Partial least squares discriminant analysis. C) Variable importance in projection plot.

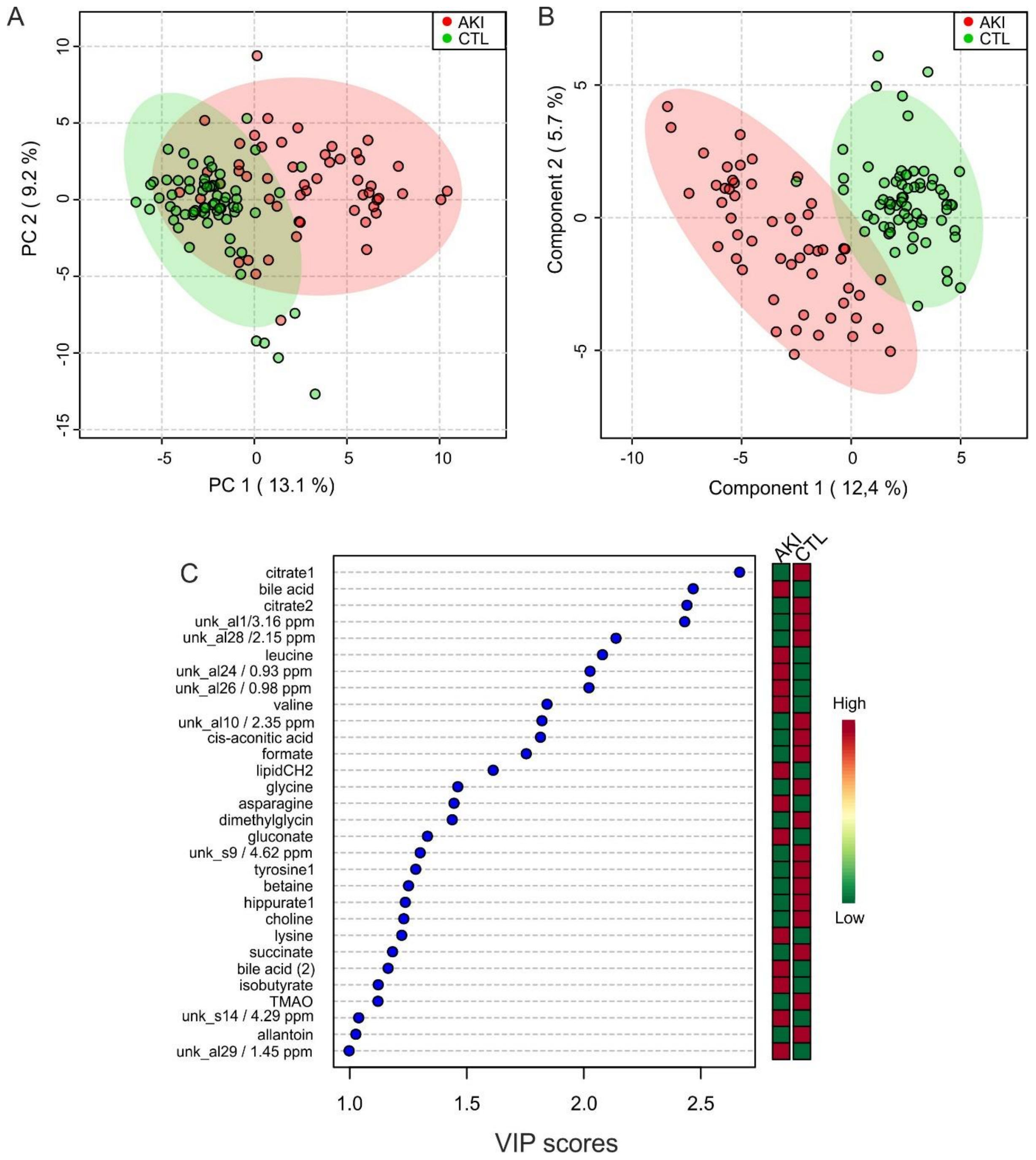

Figure S5. Same as Figure S4, with neonatal spectra excluded. A) Principal component analysis. B) Partial least squares discriminant analysis. C) Variable importance in projection plot.

Table S1. Partial least squares discriminant analysis of healthy control group vs. hospitalized patients without AKI (Non-AKI): Variable importance in projection (VIP) values.

**Bucket**	**VIP**
Lactose/unk_s3 / 5.25 ppm	2.3
Mannitol-1	2.1
*Mannitol-2**	1.7
*Mannitol-3**	1.8
*mannitol-4**	1.8
*Mannitol-5**/guanidoacetic acid	1.6
Mandelic acid /phenyl-acetylglycine/phenylalanine	2.0
Glucose/taurine	1.9
HPHPA/phenol	1.9
glutamine	1.9
unk_a9 / 7.47 ppm	1.8
3-aminoisobutyrate	1.7
Hippurate-4*	1.7
creatinine	1.6
unk_s2 / 5.35 ppm	1.6
Indoxylsulfate-3*	1.5
Indoxylsulfate-4*	1.6
glycolic acid	1.6

*Mannitol, hippurate and indoxylsulfate have more than one resonance. Due to overlap with resonances of other compounds the corresponding buckets do not behave in exactly the same way in multivariate analysis. All buckets of the respective compounds were omitted from further analysis.
